# PB.33: Vacuum-assisted biopsy for lesions of uncertain malignant potential: further evidence of its suitability as a safe alternative to surgery

**DOI:** 10.1186/bcr3533

**Published:** 2013-11-08

**Authors:** D Tzias, B Bhaludin, LS Wilkinson, S George, H Gay

**Affiliations:** 1 St George's Hospital and South West London Breast Screening Service, London, UK

## Introduction

Increasing evidence supports vacuum-assisted biopsy (VAB) instead of surgical excision for the management of lesions of uncertain malignant potential (B3). This followed reports of up to 34% upgrade to malignancy on excision. In January 2012, regional guidance was adopted to clarify the options for managing such lesions. In our department, we have used similar local guidelines since 2011. We report our analysis of the management of B3 lesions before and after the introduction of VAB.

## Methods

All B3 lesions were identified retrospectively, using our screening database, before (1 January 2008 to 31 December 2009) and after (1 January 2011 to 31 December 2012) VAB use. Final pathological diagnosis following surgical excision or VAB was recorded for both groups. The findings of annual surveillance mammography were also recorded for lesions which remained B3 but were radiologically excised following VAB.

## Results

Before the use of VAB there were 94 B3 lesions, 80 (85%) of which underwent surgical excision. After the use of VAB there were 85 B3 lesions, of which 17 (20%) went on to have diagnostic surgery. The percentage of upgrade to malignancy following further sampling was equal in both groups, at 18%. Sixteen of 26 lesions, which remained B3 following VAB excision, had surveillance mammography, none of which demonstrated suspicious findings.

## Conclusion

Our study provides further evidence that VAB is a safe alternative to surgery for the management of B3 lesions, with identical upgrade rates and reassuring follow-up results, following its use in our department.

## References

[B1] Rajan S Shaaban AM Dall BJ Sharma N New patient pathway using vacuum-assisted biopsy reduces diagnostic surgery for B3 lesions Clin Radiol 2012 15 3 244 9 10.1016/j.crad.2011.09.002 22014554

[B2] Tennant SL Evans A Hamilton LJ James J Lee AH Hodi Z Ellis IO Rakha EA Wilson AR Vacuum-assisted excision of breast lesions of uncertain malignant potential (B3) - an alternative to surgery in selected cases Breast 2008 15 6 546 9 10.1016/j.breast.2008.08.005 18829318

[B3] Lieske B Ravichandran D Alvi A Lawrence DA Wright DJ Screen-detected breast lesions with an indeterminate (B3) core needle biopsy should be excised Eur J Surg Oncol 2008 15 12 1293 8 10.1016/j.ejso.2007.11.005 18162359

[B4] Wilkinson LS Wells C Teh W Desai A Wilson R The management of indeterminate breast lesions - a clinicians guide. London region quality assurance reference centre. Guidance on management of indeterminate breast lesions 2012

